# Advantages of Tailored Isotretinoin Treatment in Moderate to Severe Acne: Real-Life Data

**DOI:** 10.3389/fphar.2021.733526

**Published:** 2021-11-12

**Authors:** Nevena Skroza, Ersilia Tolino, Veronica Balduzzi, Nicoletta Bernardini, Alessandra Mambrin, Anna Marchesiello, Federica Marraffa, Giovanni Rossi, Salvatore Volpe, Ilaria Proietti, Concetta Potenza

**Affiliations:** Dermatology Unit “Daniele Innocenzi”, “A. Fiorini” Hospital, Terracina, Italy

**Keywords:** isotretinoin, efficacy, safety, acne severity, gender

## Abstract

This retrospective single-center study analyzes the efficacy and safety of isotretinoin for the treatment of moderate to severe acne in real-life clinical practice, particularly with regard to acne severity, isotretinoin cumulative dosage, and patients’ gender. The results suggest the opportunity of an early isotretinoin systemic treatment in patients affected by moderate acne and emphasize the importance of an appropriate dose adjustment in order to minimize adverse events.

## Introduction

Isotretinoin (13-cis-retinoic acid, a derivative of vitamin A) was approved by the Food and Drug Administration (FDA) for the systemic treatment of nodulocystic acne in 1982. At first, it was reserved to patients affected by severe nodulocystic or conglobate acne. Nowadays, it is also indicated for patients with moderate acne, resistant to oral antibiotics associated with topical therapy (Cunliffe et al., 1997; Layton, 2010). Isotretinoin is the only therapy acting on four acne principal pathogenic mechanisms: reducing sebaceous glands’ dimensions and excretion rate, normalizing infundibular keratinization, decreasing drastically *Cutibacterium acnes* population, and, consequently, inflammation (Nast et al., 2010; Bagatin and Costa, 2020; Landis, 2020).

Moreover, it modulates inflammation working on cellular targets and normalizing innate immune responses mediated by Toll-like receptors, which are hyperactive in acne (Falcon et al., 1986; Coates et al., 1997). In the early phase of therapy, isotretinoin induces apoptosis of sebocytes by activation of tumor suppressor genes, downregulation of genes implicated in lipid metabolism, and upregulation of genes which codify for collagen and fibronectin (Dispenza et al., 2012).

Most common oral isotretinoin adverse events are cheilitis, xerosis, eczema, and blefaroconjuntivitis. They are predictable, dose-dependent, and easily manageable with emollients, lubricants, and moisturizing agents (Nelson et al., 2009; Layton, 2010).

Mild increase of transaminases is also frequent, but reversible, with a fast return to pre-treatment levels following isotretinoin discontinuation (Rademaker, 2013). Other reported adverse events are headache, intracranial hypertension, depression, myalgias, arthralgias, and teratogenicity (Jones et al., 1983; Hull and Demkiw-Bartel, 2000). A transient clinical worsening of acne occurring in 6% of patients has also been reported, during the first month of treatment (Demirci Saadet, 2021).

## Materials and Methods

The retrospective single-center study was carried on patients affected by moderate or severe acne who had been treated with oral isotretinoin from January 2016 to December 2020. Isotretinoin daily dosages were determined according to body weight, and all cumulative doses at the end of the first treatment cycle were estimated. Therapy was tailored on personal basis, starting with lower doses to the maximum tolerated dosage. The Global Acne Grading System (GAGS) score and Assessment of Quality of Life (AQoL) questionnaire were calculated at baseline and at the end of the first treatment cycle, in order to evaluate overall improvement. Adverse events occurring during the treatment period were registered in order to evaluate tolerability. Primary endpoints of the study were isotretinoin efficacy (estimated as reduction of GAGS score and increase of AQoL value) at the end of the first treatment cycle; isotretinoin efficacy related to its cumulative dose; isotretinoin tolerability; and recurrence rate after the end of the first treatment cycle. The patients were observed from the end of the first cycle until the end of the study. We performed pelvic echography and hormonal profile for all the female subjects; no alterations that could interfere with the treatment were found. Secondary endpoints were comparisons of isotretinoin efficacy and tolerability in male and female patients at the end of the first treatment cycle.

Quantitative parameters were analyzed by calculating the mean, the median, and the standard deviation. Also, pre- and post-treatment absolute variations (delta) of these quantitative parameters were compared. Statistical analyses were made using a software program (MedCalc). The results were analyzed using the chi-squared test, Student’s *t*-test, the analysis of variance (ANOVA) test, the Mann–Whitney *U*-test, and the Wilcoxon test. The statistical significance level was 95% (*p* < 0.05).

## Results

A total of 140 patients (mean age 22 years, age range 16–52 years) were enrolled; 46% of them were male (65 patients, mean age 20 years, age range 16–33 years), and 54% were female (75 patients, mean age 24 years, age range 17–52 years). At baseline, 88 patients (63%) were affected by moderate papulopustular acne previously resulted resistant to oral antibiotics: 37 of them were female (42%) and 51 were male (58%). Instead, 52 patients (37%) were affected by severe nodulocystic acne: 28 (54%) females and 24 (46%) males. The average duration of first therapy cycle was 5.6 months (23 weeks). The average daily dosage was 0.41 mg/kg (range 0.2–0.6 mg/kg): 0.39 mg/kg in males (range 0.2–0.6 mg/kg) and 0.43 mg/kg in females (range 0.2–0.6 mg/kg). The average cumulative dosage was 75,36 mg/kg: 72.8 mg/kg in males and 78,2 mg/kg in females.

At baseline, the mean GAGS score was 27 (95% CI 26–28) and mean AQoL value was 60 (95% CI 50–60). At the end of the first treatment cycle, the mean GAGS score was 2 (95% CI 2-3) and mean AQoL value was 90 (95% CI 89-90). Thus, the improvement was assessed as a decrease of GAGS score by 93% and increase of AQoL value by 50%. In the severe acne group, the mean delta GAGS score was 30 (95% CI 30-30), while in the moderate acne group, it was 22 (95% CI 20–23) (*p* < 0.0001). In the severe acne group, the mean delta AQoL value was 59 (95% CI 56–63), while in the moderate acne group, it was 66 (95% CI 65-66) (*p* < 0.0001). GAGS score improvement was significantly greater in patients treated with a medium/high to high isotretinoin cumulative dosage (100–120 mg/kg, >120 mg/kg) than in patients treated with a medium/low to low cumulative dosage (80–100 mg/kg, <80 mg/kg). No significant differences in isotretinoin efficacy were observed between males and females. Otherwise, any significant relationship between AQoL value improvement and isotretinoin cumulative dosage was not observed. Finally, no significant differences were observed in acne recurrence between groups treated with medium/high and high isotretinoin cumulative dosages (*p* > 0.005).

During isotretinoin treatment, 16 (11%) patients developed laboratory parameter abnormalities: 7 (44%) males and 9 (56%) females. In particular, 10 (7%) patients developed hypertransaminasemia and 5 (4%) patients developed hypercholesterolemia. All the laboratory abnormalities were of low grade and reverted with isotretinoin discontinuation.

Most of patients (76%, 55 males and 52 females) presented at least one adverse event: 72 (51%) patients (40 males and 32 females) presented only one adverse event, 30 (21%) patients (18 females and 12 males) presented two adverse events, and 5 (4%) patients (2 males and 3 females) presented four adverse events. In the low isotretinoin cumulative dosage group, 60 (77%) patients presented adverse events: 45 (57%) patients presenting only one, 13 (17%) patients two, and 2 (3%) patients three adverse events. In the medium/low isotretinoin cumulative dosage group, 19 (65%) patients presented adverse events: 16 (55%) patients presenting only one and 3 (10%) patients two adverse events. In the medium/high isotretinoin cumulative dosage group, 13 (87%) patients presented adverse events: 8 (53%) patients presenting only one and 5 (34%) patients two adverse events. In the high isotretinoin cumulative dosage group, 15 (83%) patients presented adverse events: 3 (17%) patients presenting only one, 9 (50%) patients two, and 3 (17%) patients three adverse events. The most common adverse events were cheilitis (60 patients: 33 males and 27 females) and xerosis (62 patients: 31 males and 31 females). Other adverse events observed were epistaxis, headache, tachycardia, decrease in visual acuity, chalazion, scalp dermatitis, somnolence, and keratitis.

The demographic and clinical characteristics of the patients are summarized in Table 1.

**TABLE 1:** Cumulative dose.

	Cumulative dose			
—	<80 mg/kg	80–100 mg/kg	100–120 mg/kg	>120 mg/kg
Patients, *n*	78	29	15	18
Age, mean (range)	23 (16–52)	22 (17–31)	20 (18–30)	20 (17–20)
Female, *n* (%)	33 (42)	12 (41)	67 (10)	50% (9)
Male, *n* (%)	45 (58)	17 (59)	5 (33)	9 (50)
GAGS, mean (range)	2 (59–38)	25 (12–33)	31 (26–35)	33 (31–36)
AQoL, mean (range)	60 (14–92)	62 (34–95)	50 (35–70)	45 (35–90)
Laboratory parameter abnormalities, *n* (%)	5 (6)	1 (3)	5 (33)	5 (28)
Hypertransaminasemia, *n* (%)	4 (5)	0	2 (13)	4 (22)
Thrombocytopenia, *n* (%)	1 (1)	0	0	0
Hypercholesterolemia, *n* (%)	0	1 (3)	3 (20)	1 (6)
Adverse effects, *n* (%)	60 (77)	19 (65)	13 (87)	15 (83)
Cheilitis, *n* (%)	33 (42)	10 (34)	5 (33)	10 (56)
Xerosis, *n* (%)	33 (42)	10 (34)	9 (60)	8 (44)
Relapse, *n* (%)	7 (9)	1 (3)	3 (20)	4 (22)

## Discussion

This study puts in evidence that oral isotretinoin efficacy, estimated in terms of GAGS score increase, results to be significantly greater in patients affected by severe acne than in patients affected by moderate acne. Nevertheless, advanced quality of life (increase of AQoL value) due to oral isotretinoin treatment was significantly greater in patients affected by moderate acne than in patients affected by severe acne.

In terms of different doses, no statistically significant differences were observed in GAGS score improvement, AQoL value increase, and acne recurrence rate between patients treated with a medium/high cumulative dosage (100–120 mg/kg) and patients treated with a high cumulative dosage (>120 mg/kg). It confirms that the isotretinoin cumulative dosage lower than that recommended (120 mg/kg) does not determine a higher recurrence probability, as reported by Rademaker (2013).

The sample size was greater in the group of patients receiving low isotretinoin cumulative dosage because this group also encompassed all patients who required dose adjustment because of the development of adverse events. In addition, the results showed how adverse events are dose-dependent: low doses are more tolerable but require longer periods of treatment to obtain the same clinical improvement. A limit of the study can be the small sample size; furthermore, we did not perform a multivariate analysis to calculate the relative impact of demographic and clinical variables.

In summary, our study highlights that, in the era of antibiotic resistance, an early systemic isotretinoin treatment should be taken in consideration in those patients affected not only by severe but also by moderate acne. Furthermore, our data show that there is not any clinical advantage of an isotretinoin cumulative dosage higher than 120 mg/kg, which is conversely associated with an increased risk of adverse events that could result in compliance reduction. In conclusion, given the chronic-relapsing course of acne, it is crucial to adopt a tailored therapeutic approach, taking advantage of dose adjustment in order to gain good clinical outcomes, minimize side effects, and improve patient compliance and adherence (Zomerdijk et al., 2014; Choi et al., 2021; Clark and Cunliffe, 1995).

## Data Availability

The raw data supporting the conclusions of this article will be made available by the authors, without undue reservation.
